# Mst1 promotes mitochondrial dysfunction and apoptosis in oxidative stress-induced rheumatoid arthritis synoviocytes

**DOI:** 10.18632/aging.103643

**Published:** 2020-07-21

**Authors:** Yingjie Wang, Qi Yang, Songpo Shen, Linjie Zhang, Yongbo Xiang, Xisheng Weng

**Affiliations:** 1 Department of Orthopedic Surgery, Peking Union Medical College Hospital, Peking Union Medical College and Chinese Academy of Medical Science, Beijing 100730, China; 2 Department of Orthopedic Surgery, First Hospital of Harbin, Harbin 150010, China; 3 Department of Orthopedic Surgery, Beijing Tongren Hospital, Capital Medical University, Beijing 100730, China

**Keywords:** synoviocytes, RA, Mst1, AMPK, Sirt1

## Abstract

In this study, we investigated the role of macrophage stimulating 1 (Mst1) and the AMPK-Sirt1 signaling pathway in the oxidative stress-induced mitochondrial dysfunction and apoptosis seen in rheumatoid arthritis-related fibroblast-like synoviocytes (RA-FLSs). Mst1 mRNA and protein expression was significantly higher in hydrogen peroxide (H_2_O_2_)-treated RA-FLSs than untreated controls. H_2_O_2_ treatment induced the mitochondrial apoptotic pathway by activating caspase3/9 and Bax in the RA-FLSs. Moreover, H_2_O_2_ treatment significantly reduced mitochondrial membrane potential and mitochondrial state-3 and state-4 respiration, but increased reactive oxygen species (ROS). Mst1 silencing significantly reduced oxidative stress-induced mitochondrial dysfunction and apoptosis in RA-FLSs. Sirt1 expression was significantly reduced in the H_2_O_2_-treated RA-FLSs, but was higher in the H_2_O_2_-treated Mst1-silenced RA-FLSs. Pretreatment with selisistat (Sirt1-specific inhibitor) or compound C (AMPK antagonist) significantly reduced the viability and mitochondrial function in H_2_O_2_-treated Mst1-silenced RA-FLSs by inhibiting Sirt1 function or Sirt1 expression, respectively. These findings demonstrate that oxidative stress-related upregulation and activation of Mst1 promotes mitochondrial dysfunction and apoptosis in RA-FLSs by inhibiting the AMPK-Sirt1 signaling pathway. This suggests the Mst1-AMPK-Sirt1 axis is a potential target for RA therapy.

## INTRODUCTION

Rheumatoid arthritis (RA) is a chronic and progressive systemic autoimmune disease that affects bones and joints [1]. The pathogenesis of RA involves infiltration of inflammatory cells into the joints, degeneration of the synoviocytes and abnormal synovial hyperplasia caused by infiltration of the fibroblast-like synoviocytes (FLSs) that eventually leads to destruction of the cartilage and bone [2]. Anti-inflammatory and immunomodulatory drugs are the primary modes of treating RA [3].

Oxidative stress-induced cellular apoptosis plays an integral role in RA pathogenesis [4]. Excessive ROS production and metabolic alterations promote senescence of the synoviocytes as well as excessive proliferation of the fibroblasts [5]. Moderate activation of the fibroblasts promote regeneration of the synoviocytes by producing paracrine cell growth factors such as vascular endothelial growth factor (VEGF), fibroblast growth factor (FGF) and platelet derived growth factor (PDGF), whereas, uncontrolled oxidative stress promotes phenotypic changes in the fibroblasts, thereby resulting in collagen deposition and subchondral bone erosion [6]. Chronic oxidative stress also triggers synoviocyte cell death causing loss of bone tissue and fibrosis [7]. Therefore, reducing oxidative stress and synoviocyte apoptosis is critical for suppressing RA development and progression.

Macrophage stimulating 1 (Mst1) is a protein kinase that promotes apoptosis through phosphorylation of Forkhead box O (FOXO) transcription factors [8]. Mst1 is part of the Hippo signaling pathway, which controls cellular proliferation and tissue growth. Mst1 activation is associated with oxidative stress-induced cellular apoptosis in cardiomyocytes [9] and endothelial cells [10]. During cardiac ischemia reperfusion injury, Mst1 interacts with Bcl2 and activates the Bax-mediated mitochondrial pathway of apoptosis [11]. In acute cerebral damage, Mst1 inhibits MAPK-ERK signaling pathway, thereby suppressing the expression of anti-apoptotic genes [12]. During inflammatory response, Mst1 induces apoptosis by reducing mitochondrial membrane potential through phosphorylation of c-Jun N-terminal kinase [13].

Sirtuin 1 (Sirt1) is a NAD^+^-dependent histone/protein deacetylase that regulates cellular metabolism, senescence or aging, survival, stress resistance, and oxidative stress [14]. Sirt1 induces the expression of several antioxidant proteins, such as GSH, SOD, and GPX [15]. Sirt1 also inhibits oxidative stress-related signaling pathways, such as NF-κB or JNK. Sirt1 overexpression reduces the oxidative stress-induced apoptosis in the cartilage tissue by modulating BMP2 activity [16]. Moreover, Sirt1 regulates Mst1/YAP2 activation in hepatocellular carcinoma cells, thereby modulating tumor growth and progression [17]. In this study, we investigated the relationship between Sirt1 and Mst1 in oxidative stress-induced synoviocyte apoptosis with implications for RA therapy.

## RESULTS

### Mst1 mediates oxidative stress-induced caspase-3 dependent apoptosis in the RA-FLSs

**Figure 1:**
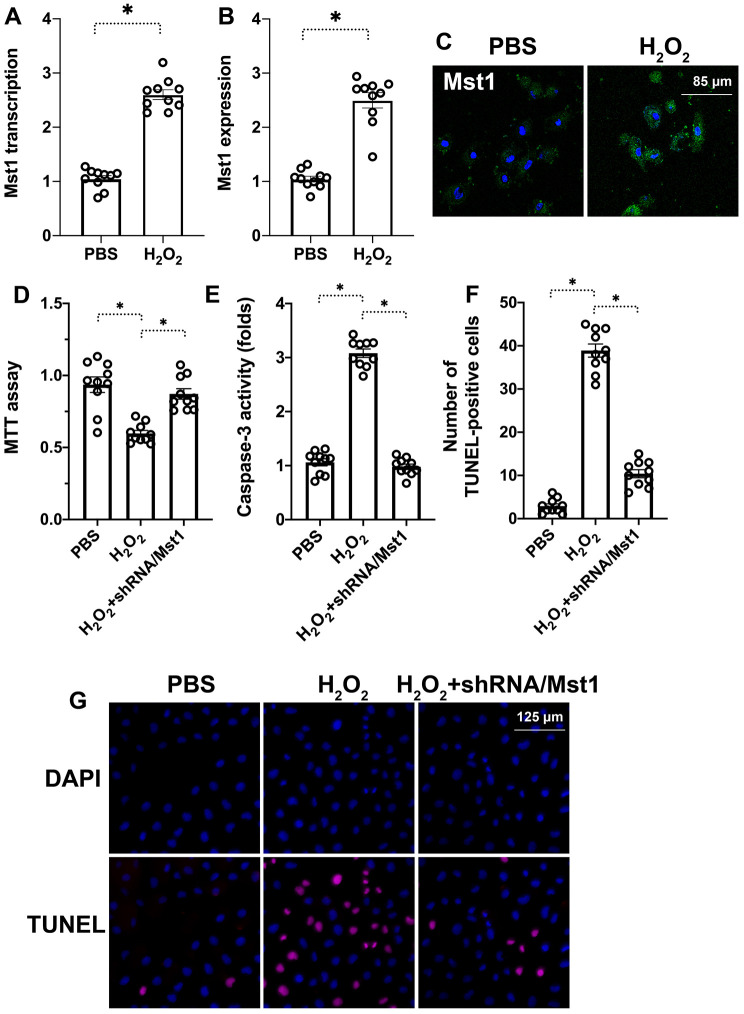
**Mst1 promotes apoptosis in oxidative stress-induced RA-FLSs.** (**A**) QRT-PCR analysis shows Mst1 mRNA levels in the control and H_2_O_2_-treated RA-FLSs. (**B**) Representative immunofluorescence images show Mst1 protein expression in control and H_2_O_2_-treated RA-FLSs (**C**) Quantitative estimation of relative Mst1 protein levels in control and H_2_O_2_-treated RA-FLSs based on the immunofluorescence assay. (**D**) MTT assay results show the viability of control and Mst1 knockdown RA-FLSs treated with or without H_2_O_2._ (**E**) ELISA assay results show caspase-3 activity in the control and H_2_O_2_-treated RA-FLSs. (**F**) Representative images show TUNEL staining of the control and H_2_O_2_-treated RA-FLSs. (**G**) Quantification of percent TUNEL-positive (apoptotic) cells in the control and H_2_O_2_-treated RA-FLSs. Note: RA-FLSs were treated with 0.3 mM H_2_O_2_ for 6 h; *P<0.05.

We induced oxidative stress in RA-FLSs using hydrogen peroxide (H_2_O_2_) and analyzed its effects on Mst1 expression. QRT-PCR analysis demonstrated that Mst1 mRNA levels were significantly higher in the H_2_O_2_-treated RA-FLSs compared to the untreated controls (Figure 1A). Furthermore, immunofluorescence assays showed that Mst1 protein levels were significantly upregulated in the H_2_O_2_-treated RA-FLSs compared to the untreated controls (Figure 1B, 1C). These results demonstrate that oxidative stress upregulates Mst1 expression in the RA-FLSs.

Next, we knocked down Mst1 in RA-FLSs using Mst1-specific shRNA (sh-Mst1) and investigated the oxidative stress response of Mst1-silenced RA-FLSs. MTT assay results demonstrated that the viability of H_2_O_2_-treated Mst1-silenced RA-FLSs was significantly higher compared to the viability of H_2_O_2_-treated RA-FLSs (Figure 1D). Furthermore, ELISA assays showed that caspase-3 activity was significantly lower in the H_2_O_2_-treated Mst1-silenced RA-FLSs compared to the H_2_O_2_-treated RA-FLSs (Figure 1E). TUNEL assay showed that apoptotic rate of H_2_O_2_-treated RA-FLSs was significantly higher compared to untreated controls and H_2_O_2_-treated Mst1-silenced RA-FLSs (Tunel-positive cells: 38% vs. 3% vs. 10%; Figure 1F–1G). This suggests that Mst1 mediates oxidative stress-induced apoptosis in the RA-FLSs.

### Mst1 activation induces mitochondrial dysfunction in oxidative stress-induced RA-FLSs

**Figure 2:**
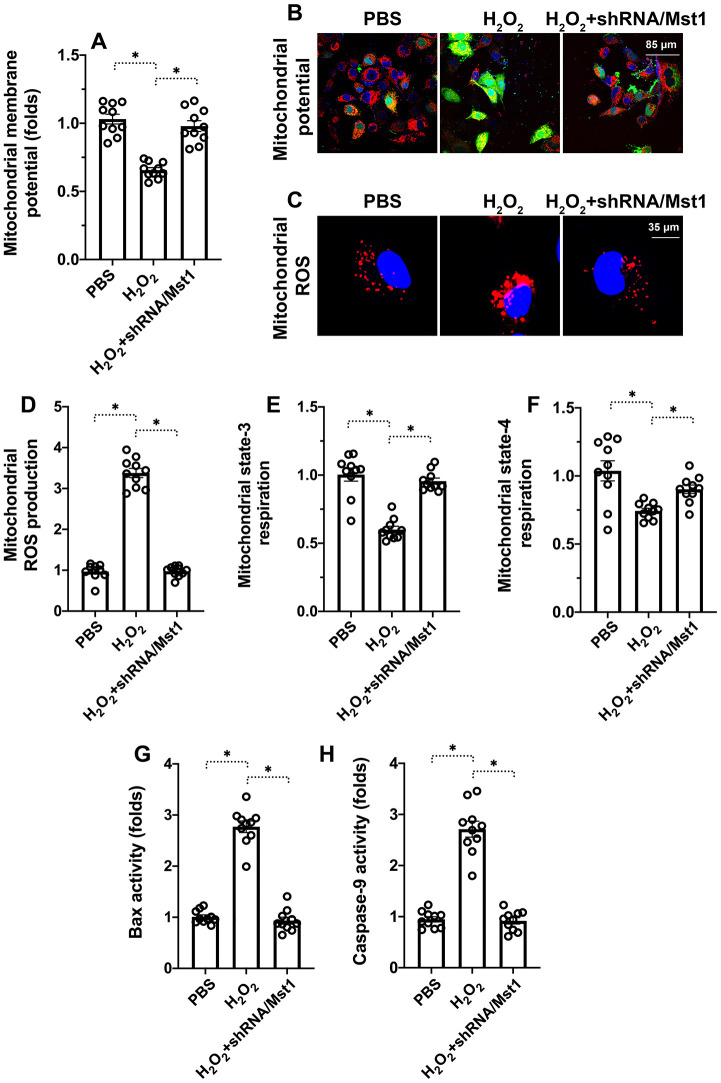
**Mst1 induces mitochondrial dysfunction in oxidative stress-induced RA-FLSs.** (**A**, **B**) Representative images show JC-1 staining to determine mitochondrial membrane potential in control and H_2_O_2_-treated RA-FLSs. Mitochondrial potential was measured by the ratio of red-to-green JC-1 fluorescence intensity. (**C**, **D**) ELISA assay results show mitochondrial state-3 and state-4 respiration rates in the control and H_2_O_2_-treated RA-FLSs. (**E**, **F**) Representative fluorescence microscopic images show DCFDA staining to determine ROS levels in the control and Mst1-silenced RA-FLSs treated with or without H_2_O_2_. ROS levels were quantified based on the DCFDA staining intensities. (**G**–**H**) ELISA assay results show caspase-9 and Bax activities in the control and H_2_O_2_-treated RA-FLSs. Note: RA-FLSs were treated with 0.3 mM H_2_O_2_ for 6 h; *P<0.05.

Mitochondria play a significant role in the regulation of cellular apoptosis and oxidative stress [18, 19]. Therefore, we investigated if Mst1 promotes apoptosis in H_2_O_2_-treated RA-FLSs by triggering mitochondrial dysfunction. Mitochondrial membrane potential was significantly reduced in the H_2_O_2_-treated RA-FLSs compared to untreated controls and H_2_O_2_-treated Mst1 knockdown RA-FLSs based on JC-1 staining (Figure 2A, 2B). ELISA assay showed that state-3 and state-4 mitochondrial respiration was significantly decreased in the H_2_O_2_-treated RA-FLSs compared to the untreated controls, but remained higher in the H_2_O_2_-treated Mst1 knockdown RA-FLSs (Figure 2C, 2D). ROS levels were significantly higher in the H_2_O_2_-treated RA-FLSs compared to the untreated controls, but lower in the H_2_O_2_-treated Mst1 knockdown RA-FLSs based on DCFDA staining (Figure 2E, 2F). These results suggest that oxidative stress induces mitochondrial dysfunction by activating Mst1.

Finally, we analyzed the status of the mitochondrial apoptotic pathway using ELISA assays. Caspase-9 and Bax activities were significantly increased in the H_2_O_2_-treated RA-FLSs compared to the untreated controls, but were significantly lower in the H_2_O_2_-treated Mst1 knockdown RA-FLSs (Figure 2G, 2H). This suggests that Mst1 promotes oxidative stress-mediated mitochondrial apoptosis in RA-FLSs.

### Mst1 inhibits Sirt1 expression in oxidative stress-induced RA-FLSs

**Figure 3:**
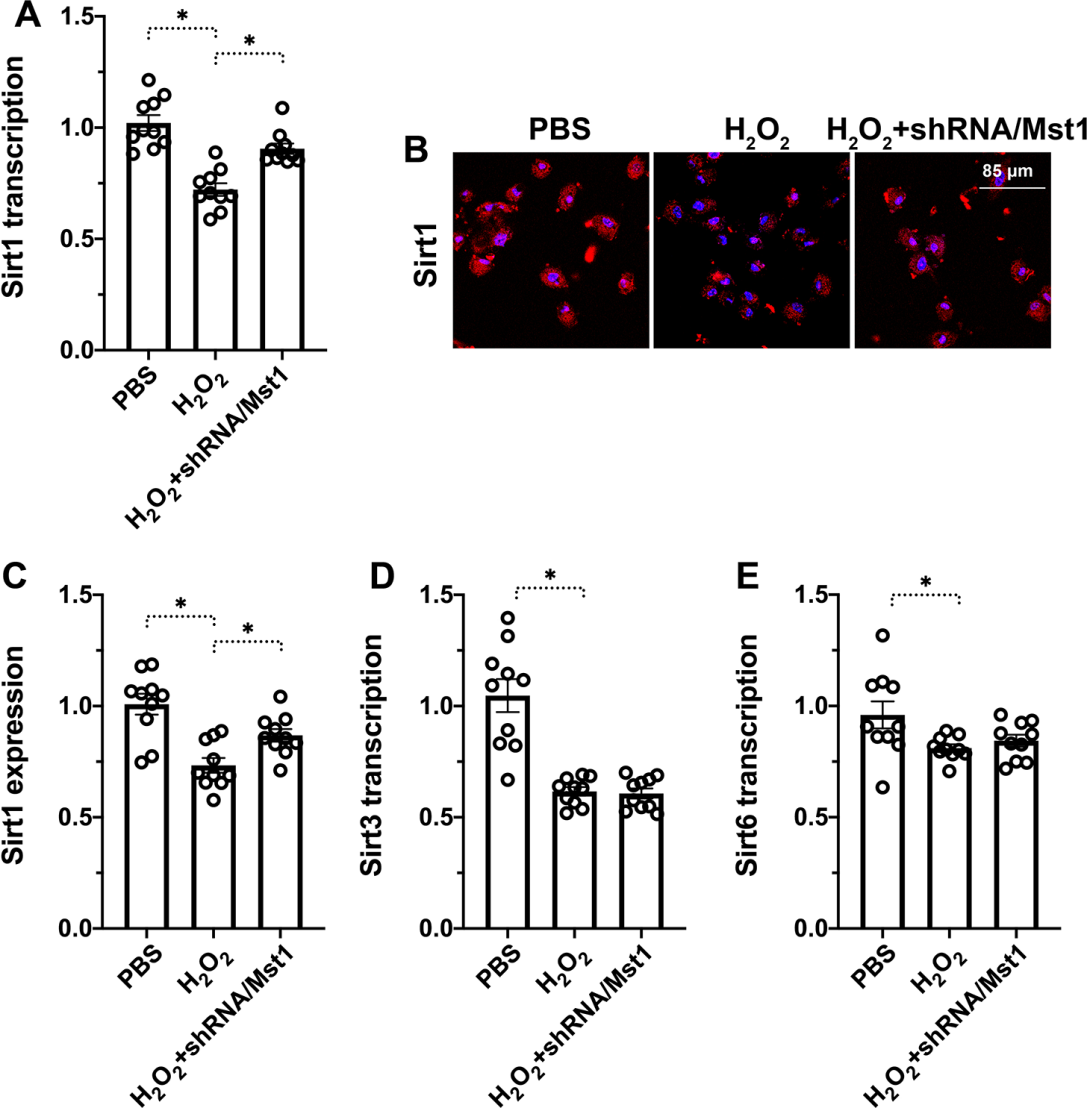
**Mst1-dependent Sirt1 downregulation in oxidative stress-induced RA-FLSs.** (**A**) QRT-PCR assay shows Sirt1 mRNA levels in control and H_2_O_2_-treated RA-FLSs. (**B**, **C**) Immunofluorescence staining shows Sirt1 protein levels in the control and Mst1-silenced RA-FLSs treated with or without H_2_O_2_. (**D**, **E**) QRT-PCR assay shows Sirt3 and Sirt6 mRNA levels in the control and H_2_O_2_-treated RA-FLSs. Note: RA-FLSs were treated with 0.3 mM H_2_O_2_ for 6 h; *P<0.05.

Next, we explored the downstream effectors of Mst1 that regulate mitochondrial dysfunction and apoptosis in RA-FLSs. Towards this, we investigated the functional status of Sirt1, which has been previously reported to be associated with cartilage apoptosis [16]. QRT-PCR analysis showed that Sirt1 mRNA levels were significantly reduced in H_2_O_2_-treated RA-FLSs compared to the untreated controls, but remained higher in the H_2_O_2_-treated Mst1 knockdown RA-FLSs (Figure 3A). Immunofluorescence assays showed that Sirt1 protein levels were significantly reduced in the H_2_O_2_-treated RA-FLSs compared to the untreated controls, but were higher in the H_2_O_2_-treated Mst1 knockdown RA-FLSs (Figure 3B, 3C). We then measured the mRNA expression of Sirt3 and Sirt6, the other members of the Sirtuin family, and observed that Sirt3 and Sirt6 levels were downregulated in the H_2_O_2_-treated RA-FLSs as well as H_2_O_2_-treated Mst1 knockdown RA-FLSs compared to the untreated controls (Figure 3D, 3E). Taken together, our results confirm that Mst1 specifically inhibits Sirt1 expression in oxidative stress-induced RA-FLSs.

### Sirt1 regulates survival and mitochondrial function in Mst1-knockdown RA-FLSs

**Figure 4:**
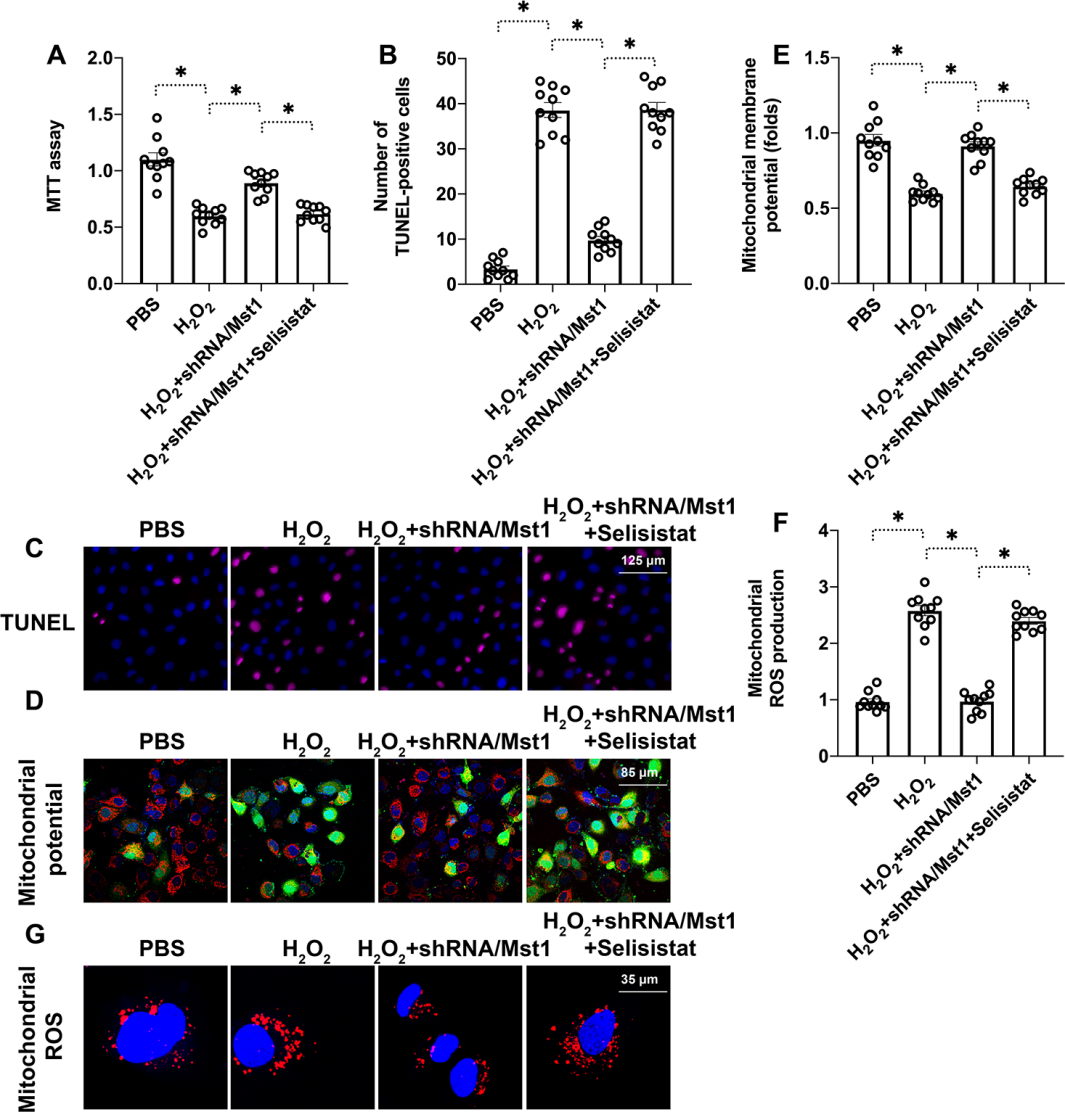
**Sirt1 inhibition abolishes the beneficial effects of Mst1 knockdown in oxidative stress-induced RAFLSs.** (**A**) MTT assay results show the viability of control and Mst1-knockdown RA-FLSs, pretreated with or without selisistat, a potent inhibitor of Sirt1. (**B**, **C**) TUNEL assay results show the apoptotic rates (percent Tunel-positive cells) in the control and Mst1-knockdown RA-FLSs, pretreated with or without selisistat, and treated with or without 0.3 mM H_2_O_2_ for 6 h. (**D**, **E**) JC-1 staining assay results show mitochondrial membrane in the control and Mst1-knockdown RA-FLSs, pretreated with or without selisistat, and treated with or without 0.3 mM H_2_O_2_ for 6 h. Mitochondrial potential was measured by the ratio of red-to-green JC-1 fluorescence intensity. (**F**–**G**) Representative fluorescence microscopic images show the DCFDA staining to determine ROS levels in the control and Mst1-knockdown RA-FLSs, pretreated with or without selisistat, and treated with or without 0.3 mM H_2_O_2_ for 6 h. ROS levels were quantified based on DCFDA staining intensity. *P<0.05.

Next, we analyzed if reduced Sirt1 expression was responsible for apoptosis and mitochondrial dysfunction in H_2_O_2_-treated RA-FLSs. We used selisistat, a potent inhibitor of Sirt1 to suppress its inhibited Sirt1 in H_2_O_2_-treated Mst1 knockdown RA-FLSs using selisistat, a potent inhibitor of Sirt1, and analyzed its effects through biochemical assays. MTT assay results showed that H_2_O_2_ treatment significantly reduced the viability of selisistat-pretreated Mst1-knockdown RA-FLSs compared to the control Mst1-knockdown RA-FLSs (Figure 4A). Tunel assay showed that the numbers of apoptotic (Tunel-positive) cells were significantly higher in the selisistat-pretreated Mst1-knockdown RA-FLSs compared to the control Mst1-knockdown RA-FLSs when treated with H_2_O_2_ (Figure 4B, 4C). Furthermore, mitochondrial membrane potential was significantly reduced (Figure 4D, 4E) and ROS levels were significantly higher (Figure 4F, 4G) in the selisistat-pretreated Mst1-knockdown RA-FLSs compared to the control Mst1-knockdown RA-FLSs upon H_2_O_2_ treatment. These results demonstrate that Sirt1 is required for survival and mitochondrial function in oxidative stress-induced Mst1-knockdown RA-FLSs.

### Mst1 reduces Sirt1 expression through inhibiting AMPK pathway

**Figure 5:**
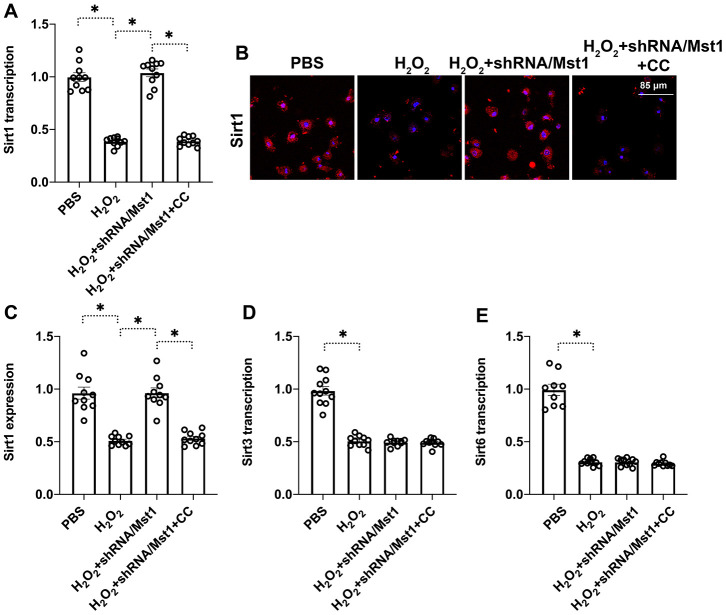
**Mst1 reduces Sirt1 expression in oxidative stress-induced RA-FLSs by inhibiting the AMPK signaling pathway.** (**A**) QRT-PCR assay results show the Sirt1 mRNA levels in the control and Mst1-knockdown RA-FLSs, pretreated with or without compound C, and treated with or without 0.3 mM H_2_O_2_ for 6 h. Compound C (CC) is an antagonist of the AMPK pathway. (**B**, **C**) Immunofluorescence staining results show Sirt1 protein levels in the control and Mst1-knockdown RA-FLSs, pretreated with or without compound C, and treated with or without 0.3 mM H_2_O_2_ for 6 h. (**D**, **E**) QRT-PCR analysis shows Sirt3 and Sirt6 mRNA levels in the control and Mst1-knockdown RA-FLSs, pretreated with or without compound C, and treated with or without 0.3 mM H_2_O_2_ for 6 h. *P<0.05.

Finally, we investigated the role of AMPK signaling pathway, a key regulator of Sirt1 stability and function, in the survival of oxidative stress-induced Mst1-knockdown RA-FLSs by using compound C (CC), the inhibitor of AMPK. QRT-PCR analysis showed that Sirt1 mRNA levels were significantly reduced in compound C-pretreated Mst1-knockdown RA-FLSs compared to the Mst1-knockdown RA-FLSs, when treated with H_2_O_2_ (Figure 5A). Immunofluorescence assays showed that Sirt1 protein levels were significantly lower in the compound C-pretreated Mst1-knockdown RA-FLSs compared to the Mst1-knockdown RA-FLSs, when treated with H_2_O_2_ (Figure 5B, 5C). QRT-PCR analysis also showed that H_2_O_2_treatment increased Sirt3 and Sirt6 levels in Mst1-knockdown RA-FLSs with or without compound pre-treatment (Figure 5D, 5E). These data demonstrate that Mst1 specifically inhibits Sirt1 expression through the AMPK pathway in the oxidative stress-induced RA-FLSs.

## DISCUSSION

In the present study, we demonstrate that oxidative stress-induced apoptosis and mitochondrial dysfunction in the synoviocytes is associated with increased Mst1 expression and dysregulated AMPK-Sirt1 signaling pathway. Oxidative stress upregulates Mst1 expression in the synoviocytes. Mst1 decreases mitochondrial respiration and mitochondrial membrane potential and increases ROS levels by suppressing AMPK-Sirt1 signaling pathway. Subsequently, Mst1 upregulation promotes Bax- and caspase-3/9-dependent mitochondrial apoptosis in oxidative stress-induced synoviocytes. Therefore, our study demonstrates that Mst1- AMPK-Sirt1 axis is a novel therapeutic target to alleviate oxidative damage-related dysfunction of the synoviocytes in RA patients.

RA is a disease caused by chronic inflammation involving recruitment of pro-inflammatory immune cells, oxidative stress and synoviocyte apoptosis, collagen accumulation and bone tissue degradation [20–23]. Therapeutic strategies that reduce free radical generation and oxidative damage relieve common symptoms such as pain and stiffness in RA patients. Drugs that reduce oxidative stress and pro-inflammatory cytokine levels, such as, Rhoifolin [24], rosmarinic acid [25], and alogliptin [26] have commonly been used for RA therapy. However, the molecular mechanisms underlying oxidative stress-mediated dysfunction and death of synoviocytes are not clear. Our study demonstrates that oxidative stress increases Mst1 expression in the synoviocytes. Mst1 is a pro-apoptotic protein that was first described in studies related to HCC progression [27]. Subsequently, Mst1 has been implicated in pathology related to cerebral ischemia-reperfusion injury [12, 28], septic cardiomyopathy [13, 29], atherosclerosis [30], neuroinflammation [31], and diabetes-related microvascular dysfunction [32]. Mst1 upregulation is associated with increased ROS and decreased ATP levels that subsequently cause cellular apoptosis [33, 34]. Thus, our study implies that Mst1 is a potential target for RA therapy.

Sirt1 is a critical component of the mitochondrial quality control machinery and is involved in the regulation of mitochondrial biogenesis, mitochondrial oxidative stress, and mitochondrial dynamics [35–37]. A recent study by Leblond et al showed that Sirt1 is associated with synovial angiogenesis, which is critical pathological phenotype observed in RA patients [38]. Besides, Sirt1 modulates osteogenic differentiation by regulating the expression of retinoic acid receptors [39]. In RA, the proliferation of synovial fibroblasts and production of pro-inflammatory cytokines is partly inhibited by the miR-22/Sirt1 signaling pathway [40]. In the present study, we found that Sirt1 expression is significantly downregulated under oxidative stress in an Mst-dependent manner. However, Sirt1 expression is high in oxidative stress-induced Mst1-silenced synoviocytes and correlated with improved mitochondrial function and viability. However, Sirt1 inhibition or AMPK inhibition abolishes the beneficial effects of the Mst1 knockdown in the oxidative stress-induced synoviocytes. Therefore, our results demonstrate a causal relationship between Mst1 activation and Sirt1 downregulation in the oxidative stress-induced synoviocytes.

In conclusion, our study demonstrates that oxidative stress-induced Mst1 upregulation in the synoviocytes promotes mitochondrial dysfunction and apoptosis by inhibiting the AMPK-Sirt1 signaling pathway. Therefore, Mst1-AMPK-Sirt1 axis is a potential target for RA therapy.

## MATERIALS AND METHODS

### RA synoviocytes and cell culture

The human rheumatoid arthritis fibroblast-like synoviocytes (RA-FLSs; C0495) were obtained from Guandao Bioengineering Co., Ltd (Shanghai, China) and maintained in DMEM medium containing 10% FBS and 1% penicillin/streptomycin. The RA-FLSs were treated with 0.3 mM hydrogen peroxide for 6 h to induce oxidative stress as previously described [41–42]. The RA-FLSs were pre-treated for 4 h with selisistat, a potent inhibitor of Sirt1, to inhibit Sirt1 activity.

### Construction of Mst1-shRNA recombinant lentivirus

Lentivirus particles containing Mst1 shRNA were prepared as previously described [43]. Briefly, the shRNA against Mst1 was inserted into the pBS-SKII-hU6 vector downstream of the hU6 promoter [44]. The shRNA expression cassette was then confirmed by DNA sequencing. Lentiviral stocks were prepared by Polyetherimide (PEI) co-transfecting HEK293T cells with the lentiviral expression and packaging plasmids, psPAX2 and pMD2.G. The supernatants were collected at 48 h and 72 h after transfection [45], and the lentiviral particles were concentrated using a 0.45 μm filter (Millipore Corp, Billerica, MA, USA). The recombinant lentiviruses were used to infect RA-FLSs using 4 μg/ml polybrene.

### Western blotting

The cells were lysed with the RIPA buffer for 2 h on ice and centrifuged at 13,200 rpm for 15 min at 4°C. The protein concentration in the supernatant was determined using the Bio-Rad protein assay kit (Bio-Rad, Hercules, CA, USA) according to the manufacturer’s protocol [46]. Equal amounts of total cellular protein lysates were subjected to SDS–PAGE electrophoresis, transferred to pre-activated polyvinylidene fluoride membranes (Millipore Corp, Billerica, MA, USA), and the membranes were blocked with 5% skimmed milk in TBST at room temperature for 1 h [47]. The membranes were then incubated overnight at 4^o^C with primary antibodies against Mst1, Sirt1 and GAPDH (Santa Cruz Biotechnology). Then, the blots were incubated with HRP-conjugated secondary antibodies at room temperature for 1 h. The blots were developed using Enhanced Chemiluminescence (ECL) solution and visualized [48]. The protein bands were quantified using the Image J software and relative amounts of proteins were estimated using GAPDH as the internal control [49].

### Immunofluorescence staining

For immunofluorescence staining, the cells were fixed with 3.7% formaldehyde in PBS and blocked with 0.1% BSA solution. The slides were then incubated overnight at 4°C with primary antibodies [50]. Then, after rinsing with PBS, the slides were incubated with Alexa Fluor® 488 goat anti-mouse and Alexa Fluor® 594 goat anti-rat secondary antibodies (Thermo Fischer, Waltham, MA) [51]. The nuclei were stained with DAPI. The stained cells were visualized under a Zeiss LSM 700 confocal laser scanning microscope (Carl Zeiss, Germany) with a 63x oil immersion objective using a dual filter set for DAPI and FITC or rhodamine. The images were captured using the ZEN software (Carl Zeiss, Germany) [52].

### TUNEL

TUNEL was performed using the *In situ* cell death detection kit (Roche, Shanghai, China) as previously described [53]. Briefly, the sections were fixed with 4% paraformaldehyde, permeabilized with buffer containing 0.5% Triton X-100 and 0.05% SDS, and then incubated with TUNEL reaction mixture in the reaction buffer [54]. The nuclei were stained with DAPI and then the specimens were visualized under microscope. TUNEL-positive cells were counted in five fields at 200x magnification for each slide and normalized to DAPI staining numbers using the Image J software [55].

### Enzyme-linked immunosorbent assay (ELISA)

For the ELISA assays, we used 2 mg of the whole cell lysate in 100 μl buffer to detect intracellular proteins, whereas for secreted proteins, 20 ml of the culture medium was concentrated using the 3 kDa cut-off Amicon Ultra-15 Centrifugal Filter (#UFC900308, EMD Millipore) to detect the concentration of targeted proteins. ELISA assays were performed according to the manufacturer’s instructions [56].

### ROS detection

The DCFDA Cellular Reactive Oxygen Species Detection Assay Kit (ab113851; Abcam) was used to estimate the reactive oxygen species (ROS) levels in cells [57]. Briefly, 2.5 x 10^4^ cells per well were seeded in dark, clear bottom 96-well microplates and allowed to adhere overnight. Then, the cells were incubated with 25 μM DCFDA for 1 h at 37°C. The fluorescence was analyzed using a fluorescent plate reader at an excitation wavelength at 485 nm and an emission wavelength at 535 nm [58].

### Quantitative real time PCR (qPCR)

Total cellular RNA was isolated using the RNeasy mini Kit (Qiagen, Hilden, Germany), purity and quantified using the Nanodrop spectrophotometer (Thermo Scientific, Waltham, MA). Then, cDNA was synthesized using MultiScribe reverse transcriptase, random primers, deoxyribose nucleoside triphosphate (dNTP) mix and RNase inhibitor (all Applied Biosystems, Foster City, CA) in a Quanta Biotech Q Cycler II according to the following cycling conditions: 2 min at 50°C; 10 min at 95°C; 40 cycles of 15 seconds at 95°C and 60 seconds at 60°C. Then, qPCR was performed using Taqman gene assays [59]. Relative gene expression was analyzed using the 2^-ΔΔCt^ method with GAPDH as the internal control.

### Statistical analysis

The data are presented as the means ± standard deviation (SD). All biochemical analyses were performed at least in triplicate. The differences between multiple groups were analyzed using one-way analysis of variance (ANOVA) and the differences between two groups were analyzed using the 2-tailed Student’s t-test, followed by Dunnett’s and Tukey’s tests. P < 0.05 was considered statistically significant. Pearson’s correlation coefficients were calculated to evaluate the association between clinical parameters. All statistical analyses were performed using the GraphPad Prism version 5.0 for Windows (GraphPad Software, San Diego, CA, USA).
